# Ecofriendly biodegradation of Reactive Black 5 by newly isolated *Sterigmatomyces halophilus* SSA1575, valued for textile azo dye wastewater processing and detoxification

**DOI:** 10.1038/s41598-020-69304-4

**Published:** 2020-07-23

**Authors:** Rania Al-Tohamy, Jianzhong Sun, Mervat F. Fareed, El-Refaie Kenawy, Sameh S. Ali

**Affiliations:** 1 Biofuels Institute, School of the Environment and Safety Engineering, Jiangsu University, Xuefu Rd. 301, Zhenjiang, 212013 China; 2 Department of Home Economic, Faculty of Specific Education, Tanta University, Tanta, Egypt; 3 Polymer Research Group, Department of Chemistry, Faculty of Science, Tanta University, Tanta, 31527 Egypt; 4 Botany Department, Faculty of Science, Tanta University, Tanta, 31527 Egypt

**Keywords:** Biological techniques, Microbiology, Environmental sciences

## Abstract

A total of seven yeast strains from 18 xylanolytic and/or xylose-fermenting yeast species isolated from the wood-feeding termite *Reticulitermes chinenesis* could efficiently decolorize various azo dyes under high-salt conditions. Of these strains, a novel and unique azo-degrading and halotolerant yeast, *Sterigmatomyces halophilus* SSA1575, has been investigated in this study. This strain could significantly decolorize four combinations of a mixture of dyes. It showed a high capability for decolorizing Reactive Black 5 (RB5) even at 1,500 mg L^−1^. The strain SSA1575 still showed a high capability for decolorizing a 50 mg L^−1^ RB5 with a salt mixing at a NaCl concentration of up to 80 g L^−1^. It also exhibited significant ability to decolorize repeated additions of dye aliquots, with a reduction in time of up to 18 h. Most of the tested carbon and nitrogen sources could significantly enhance a RB5 decolorization. However, this process was inhibited by the addition of sucrose and sodium nitrate. NADH-dichlorophenol indophenol (NADH-DCIP) reductase and lignin peroxidase were determined as the key reductase and oxidase of *S. halophilus* SSA1575. Finally, strain SSA1575, can effectively detoxify RB5 into non-toxic products. Overall, *S. halophilus* SSA1575, might be a promising halotolerant yeast valued for the treatment of various textile effluents with high salinity.

## Introduction

A variety of textile dyestuffs (approximately 10,000 synthetic dyes; 7 × 10^7^ metric tons) produced every year worldwide, posing a threat to the environmental safety^1,2^. Azo dyes represent the largest and most common used class of synthetic dyes in textile industry. However, the effluents from these industries have proven toxic, mutagenic and carcinogenic activities due to the presence of one or more azo bond^3,4^. Recently, the discharge of dye effluents into natural streams has gained great public attention owing to the serious ecological risks. Therefore, more effective and ecofriendly methods to treat industrial wastewater effluents are urgent prior to their discharge into the natural environment.

Despite the removal of azo dyes from effluents using conventional treatments, such as flocculation, membrane filtration, photocatalysis and adsorption^5–8^, microbial decolorization is a cheaper and eco-friendly accepted technique for the removal of dyes compared to conventional physical and chemical methods^3,9^. Hence, most current studies on the degradation of azo dyes are mainly focused on microbial degradation using filamentous fungi and bacteria. However, the production of aromatic amines has adverse impact on bacterial activity^10^. In comparison, fungi have been found to decolorize dyes without production of aromatic amines based on their extracellular lignin-modifying enzymes, including lignin peroxidase (LiP), manganese peroxidase (MnP) and laccase (Lac) along with other supporting enzymes^6^. However, filamentous fungi (molds) are sometimes poorly adapted in the industrial effluents due to their mycelial ageing, the large amounts of sludge produced, as well as their growth rates are usually slow when compared to most yeast species^11^. Yeasts, by contrast, have many advantages over bacteria and filamentous fungi, which present a potential capability to deal with some stringent wastewater conditions, such as a salty or acidic condition, and particularly in regard to their unique non-pathogenicity and fast growing nature^12–14^. As far as it is known, relatively limited studies are available in literature about yeast based azo dye decolorization under hypersaline conditions^15–17^.

The effluents of the wastewater textile industry are composed of a high concentration of dyes and other pollutant substances, such as a high salt concentration. For the treatment of textile effluents, it is important to use microbial strains capable of tolerating high salt concentrations greater than 3%^6^. Halophilic and salt-tolerant microorganisms have biological advantages compared to other organisms that could die or become dormant when survive under the same extreme conditions. For example, many salt-tolerant or halophilic microorganisms can keep a relatively high growth rate and metabolic efficiency under high salt conditions, since these organisms do not permit salt into their cell, while they apply the accumulation mechanism of solutes or osmolytes for living in saline habitats^6, 18^. Some salt-tolerant yeast strains, *Pichia occidentalis* G1, *Galactomyces geotrichum* GG and *Scheffersomyces spartinae* TLHS-SF1, have been confirmed as efficient decolorizing and detoxifying alternatives of azo dyes under high salinity^15–17^.

As lignin is a phenol aromatic polymer, the presence of azo dyes may induce the microbial ligninases, which have great potential in processes related to the degradation of wastewater aromatic dyes. However, the actual pathways for the biodegradation of azo dyes are still not fully understood, since the involvement of ligninase enzymes in dye degradation is "non-specific" in nature^19^. The biodegradation of azo dye Reactive Black 5 (RB5) led to the formation of dissimilar metabolic end products in two independent studies. For example, the mechanism of RB5 degradation by a yeast strain, *Trichosporon akiyoshidainum* HP-2023 led to the formation of Quinone B and phenildiazene, which were driven via phenol oxidase and peroxidase enzymes^20^. However, oxalic acid was produced as the end metabolic product of the RB5 degradation by *Aeromonas hydrophila* isolated from textile azo dye wastewater, via the cleavage of azo (–N=N–) bonds^21^. On the other hand, the application of the purified enzymes in textile dye effluents is an expensive treatment method. These enzymes often lose their catalytic potential under extreme conditions and are difficult to recover^6,22,23^. Hence, catalytically efficient ligninases-producing salt-tolerant or halophilic yeasts are required to overcome these challenges. Recently, some strains of *Sterigmatomyces halophilus*, capable of tolerating various extreme conditions, such as salt concentration, were successfully isolated and identified from the gut symbionts of a wood-feeding termite species, *Reticulitermes chinenesis* by Ali et al.^13^. This promising model insect has been reported for its capability in digesting lignin through in-situ gut symbionts that are responsible for lignin degradation^24^. These symbionts exhibited potential for some industrial applications, especially in lignin removal from lignocellulosic biomass-based biorefinery^3,24^.

Exploration of ligninase-producing yeast strains from termite gut symbionts on their capability to degrade aromatic compounds, as well as to survive under extreme conditions, such as high salt concentration is gaining attention, making the treatment of textile dyes by halotolerant yeasts a novel biodegradation approach. Therefore, this study was undertaken to identify and characterize novel halotolerant yeast species or strains isolated from the gut of *R. chinenesis* and capable of decolorizing azo dyes efficiently. Various physcio-chemical and nutritional parameters were evaluated to achieve maximum dye decolorization efficiency by *S. halophilus* SSA1575. Activities of some enzymes which might participate in dye degradation were also determined. Phytotoxicity and microbial toxicity tests were performed for estimating the toxicity of the metabolites after dye decolorization. To well understand possible degradation pathways of the target dye, the enzymatic system involved and possible metabolic intermediates analyzed, as well as related literature were studied.

## Results and discussion

### Identification and characteristics of halotolerant azo dye-decolorizing yeasts

**Figure 1:**
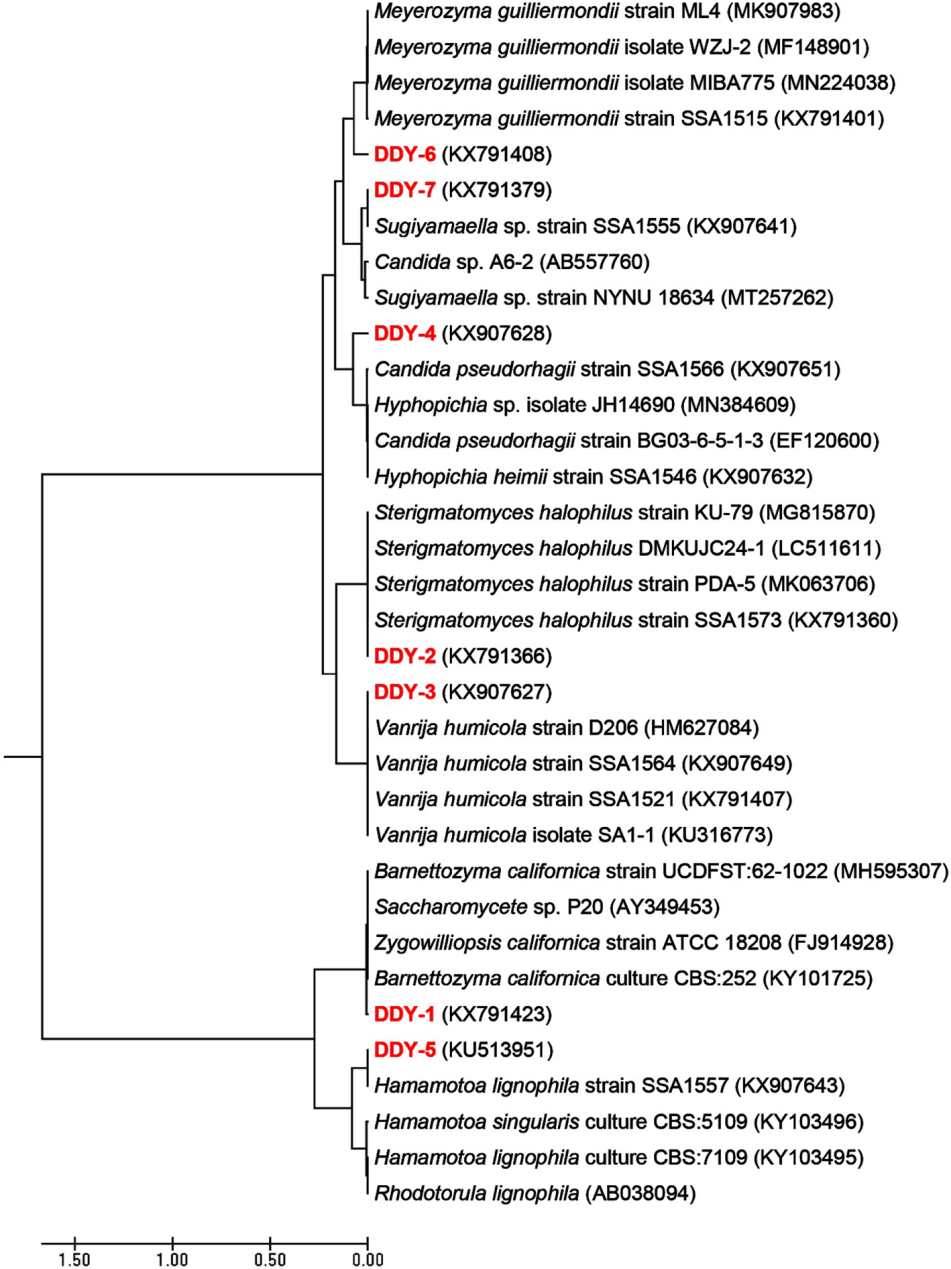
Evolutionary relationships of the seven azo-decolorizing yeast strains based on the UPGMA method as conducted by using MEGA7.

**Figure 2:**
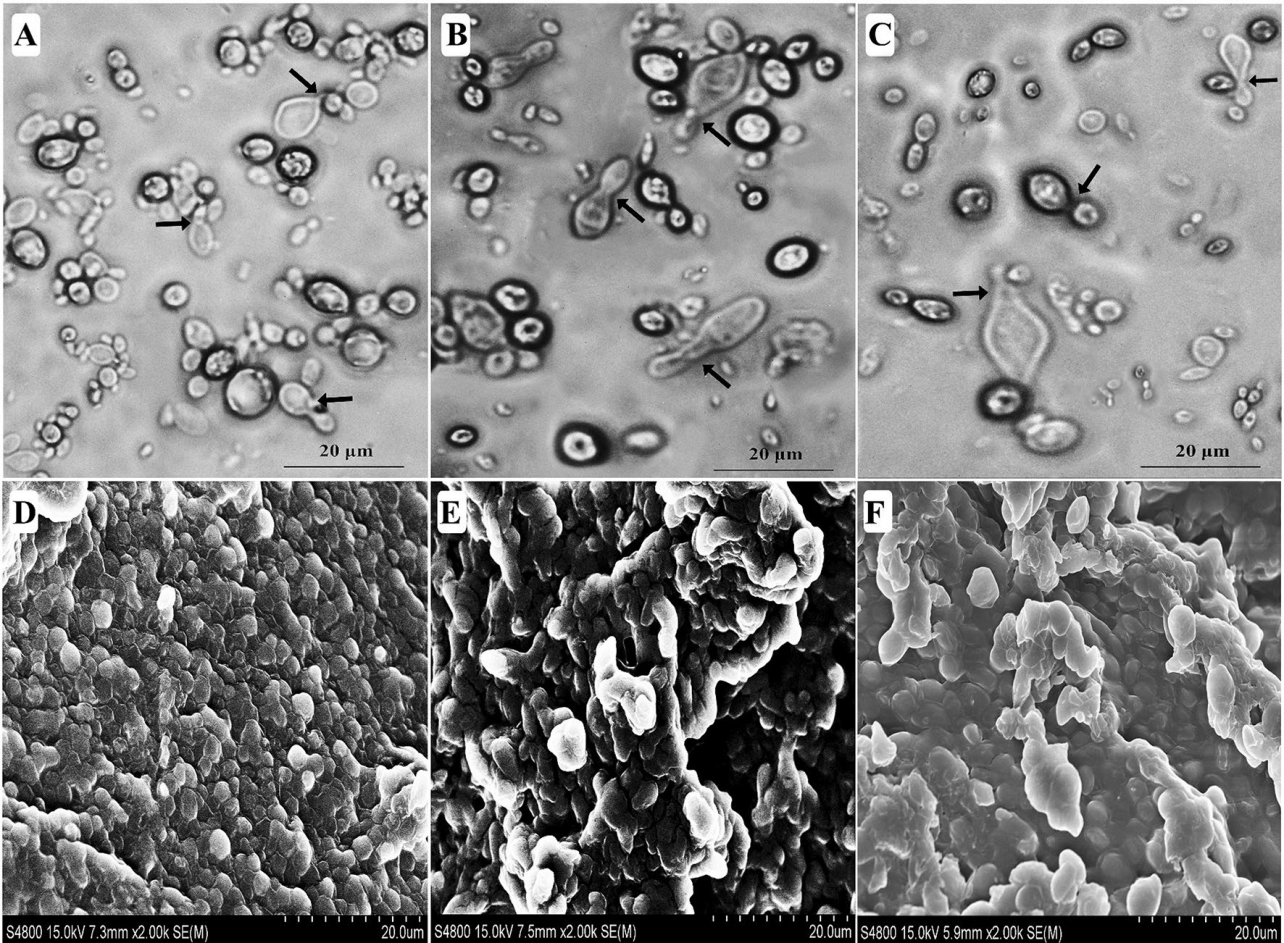
*Sterigmatomyces halophilus* SSA1575 from the wood-feeding termite *R. chinenesis*. Cell morphology of the strain SSA1575 showing spherical forms (**A**), ovoid forms (**B**) and conidia formation on interconnecting sterigmata (arrows) (**C**). Scanning electron micrographs of *S. halophilus* SSA1575 under a high salt concentration; 20 g L^−1^ NaCl (**D**), 30 g L^−1^ NaCl (**E**) and 50 g L^−1^ NaCl (**F**).

Among 18 xylanolytic and/or xylose-fermenting yeast species isolated from the wood-feeding termite *R. chinenesis*^13^, seven yeast strains could efficiently decolorize various azo dyes as confirmed from the decolorized halos observed in the Minimal Saline (MS) agar plates containing 50 mg L^−1^ of the dye tested. These yeast strains were also examined similarly in MS broth medium supplemented with 50 mg L^−1^ of the dye and incubated at 30 °C for 24 h. As given in Table 1, all yeast strains successfully decolorized RB5 with a decolorization efficiency of over 90% within 24 h of static incubation. Figure 1 depicts the neighbor-joining tree showing the phylogenetic relationship of the 26S rDNA gene sequences obtained from the seven dye decolorizing yeast strains (DDY-1 to DDY-7) alongside GenBank organisms closely related to them at NCBI (Table 2). Clearly, the strain DDY-2 presented an impressive decolorization performance of various azo dyes tested over other yeast strains (Table 1). The yeast strain DDY-2 exhibited high identity (99.34%) to *S. halophilus* strain SSA1573 (with accession number KX791360). Hence, DDY-2 was identified as *S. halophilus* strain SSA1575 with the GeneBank accession number KX791366 (Table 2 and Fig. 1). Morphologically, the cells of the newly isolated deuteromycete *S. halophilus* strain SSA1575, isolated from *R. chinenesis*, were mostly spherical (Fig. 2A) to ovoid (Fig. 2B) and produced conidia on interconnecting sterigmata (Fig. 2C). These cells did not have mycelial elements or undergo sexual recombination.

**Table 1:** Performance of tested yeast strains on decolorizing various azo dyes.

Isolate code	RB5		RR120		RB19		AzB		AS-GR	
	MD (%)	T (h)	MD (%)	T (h)	MD (%)	T (h)	MD (%)	T (h)	MD (%)	T (h)
DDY-1	93.52	24	78.23	18	93.75	18	90.80	18	81.98	15
DDY-2	98.71	12	93.46	18	94.80	15	95.88	18	96.56	21
DDY-3	95.78	21	91. 10	21	80.72	12	78.76	21	90.11	18
DDY-4	90. 82	18	88.34	24	69.12	24	91.70	24	77.98	24
DDY-5	90.76	24	75.16	24	55.89	24	88. 64	21	89.71	18
DDY-6	98.12	24	90.87	18	79. 34	18	91.09	24	69.89	24
DDY-7	93.45	18	90.06	18	92.51	24	87. 83	15	88.03	24

*RB5* Reactive Black 5, *RR120* Reactive Red 120, *RB19* Reactive Blue 19, *AzB* Azure B, *AS-GR* Acid Scarlet GR, *MD* maximum decolorization, *T* time.

**Table 2:** Molecular identification of azo-degrading yeast strains based on BLAST comparison to the GeneBank database.

Isolate code	Yeast species	Strain	Accession no	Closest relative (accession no.)	Sequence identity (%)
DDY-1	*Barnettozyma californica*	SSA1537	KX791423	*Barnettozyma californica* culture CBS:252 (KY101725)	99.45
DDY-2	*Sterigmatomyces halophilus*	SSA1575	KX791366	*Sterigmatomyces halophilus* strain SSA1573 (KX791360)	99.34
DDY-3	*Vanrija humicola*	SSA1541	KX907627	*Vanrija humicola* strain D206 (HM627084)	99.34
DDY-4	[*Candida*] *pseudorhagii*	SSA1542	KX907628	[*Candida*] *pseudorhagii* strain SSA1566 (KX907651)	88.07
DDY-5	*Hamamotoa lignophila*	SSA1567	KU513951	*Hamamotoa lignophila* strain SSA1557 (KX907643)	100
DDY-6	*Meyerozyma guilliermondii*	SSA1522	KX791408	*Meyerozyma guilliermondii* strain SSA1515 (KX791401)	87.94
DDY-7	*Sugiyamaella* sp.	SSA1592	KX791379	*Sugiyamaella* sp. strain SSA1555 (KX907641)	99.64

The majority of the yeast species *S. halophilus* was commonly isolated from marine environments^25^. However, the xylanase-producing and D-xylose-fermenting yeast *S. halophilus* was first identified from an insect gut system as per our previous study^13^. To date, to the authors’knowledge, no report has investigated the performance of *S. halophilus* on dye decolorization under high salinity. Therefore, changes in the morphological structure of strain SSA1575 under the stress of high salt conditions were observed by scanning electron microscopy (Fig. 2D–F). The growing cells with 20 g L^−1^ NaCl remained relatively normal with oval/spherical shapes. However, increasing NaCl concentration up to 50 g L^−1^ revealed obvious metamorphosis including massive compact clumps with distorted edges and irregular clumps in some yeast cells. The increasing need for bioremediation of hypersaline habitats and for biological control agents that can be used in agriculture irrigated by saline water, stimulate the search for halophilic/salt-tolerant yeasts. The cellular stress response in yeasts has a defined set of targeted cellular functions including DNA/chromatin stabilization and repair, protein chaperoning, removal of damaged proteins, and cell cycle control^26^.

As a novel yeast species that was first identified from an insect gut system, *S. halophilus* strain SSA1575 has exhibited some unique and impressive properties that are potentially valuable for biorefinery and bioremediation purposes. No report has been published on the dye decolorization using *S. halophilus*. In addition, the *S. halophilus* strain SSA1575, showed significantly higher capability on decolorizing RB5 when compared with the other yeasts that were isolated from *R. chinenesis*. Simultaneously, SSA1575 showed a high tolerance under hypersaline conditions, which suggested its valuable potential in the treatment of industrial effluents containing a wide variety of azo dyes at a high salt environment. Based on these reasons, the *S. halophilus* strain SSA1575 and RB5 were actually selected for further investigation.

### Decolorization of mixture of azo dyes

The performance of different microorganisms on the decolorization of the mixture of azo dyes was previously studied in a few reports^27–29^. While evaluating the decolorization of the mixtures of azo dyes by the *S. halophilus* strain SSA1575, it was observed that this halotolerant yeast strain was effectively decolorized mixture of azo dyes. Based on the values obtained from the American Dye Manufactuers’ Institute (ADMI) calculations, it was found that mixture I (RB5 and Reactive Black 19; RB19) was decolorized more effectively than three other mixtures (II, III and IV) at 24 h of dye addition. Mixture I showed 83.17% ADMI removal which was found to be higher than mixture II (RB5, RB19 and Reactive Red 120; RR120), mixture III (RB5, RB19, RR120 and Azure B; AzB) and mixture IV (RB5, RB19, RR120, AzB and Acid Scarlet GR; AS-GR) by 72.56%, 58.42% and 51.21%, respectively. Tamboli et al.^29^ reported that the structure variability of dye mixtures may induce a maximum production of the polyhydroxyalkanoate (PHA) synthase enzyme. PHAs are the cytoplasmic microbial inclusions, which formed in stressed conditions, i.e. in the presence of excess carbon source and when growth is restricted by the lack of other nutrients such as nitrogen, phosphorus or sulphur^30^. As the nutritional supply is reestablished, the PHA can be degraded by intracellular polymerase and subsequently metabolized to a carbon and energy source^31^.

### Dye concentration

**Figure 3:**
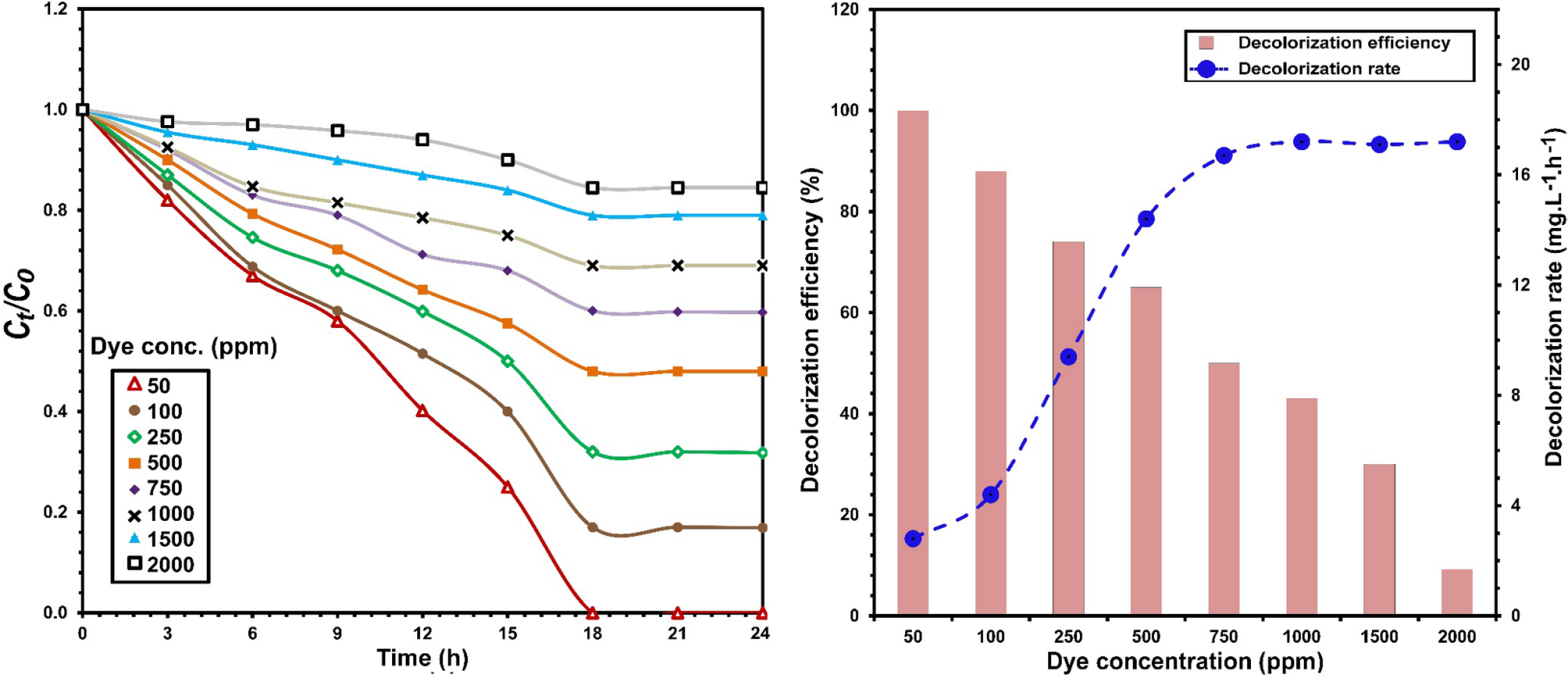
Influence of the initial concentration of RB5 on the dye decolorization performance of *S. halophilus* SSA-1575, inset with the corresponding decolorization efficiency and decolorization rate.

The effects of initial dye concentration on the decolorization efficiency of RB5 by *S. halophilus* SSA1575 are shown in Fig. 3. Clearly, the decolorization efficiency reached 100% at 50 mg L^−1^ RB5 after incubation for up to 18 h. Beside dye removal, the decolorization rate was also calculated to understand the effect of the initial dye concentration on decolorization. It has been reported that the decolorization rate may be increased with increasing dye concentration^32^. In this study, although the decolorization efficiencies decreased with increasing RB5 concentrations, the decolorization rate increased with increasing dye concentration. At a concentration of 50 mg L^−1^, the maximum decolorization rate of RB5 was obtained at 2.8 mg L^−1^ h^−1^, while it reached 17.2 mg L^−1^ h^−1^) at 2000 mg L^−1^ after 24 h. Meanwhile, maximum decolorization efficiency (> 70%) was observed at 100–500 mg L^−1^ RB5 within 24 h. Then, decolorization efficiency of RB5 decreased significantly (*p* < 0.001) with increasing dye concentration, reaching 45% at 1,000 mg L^−1^, which was probably due to the toxicity of dye at high concentrations, hence inhibiting microbial growth^33^. Dye decolorization of RB5 by *S. halophilus* SSA1575 was compared with other strains reported in literature (Table 3) and it showed a competitive decolorization performance. As the highest concentrations tested in this study are much above the dye levels in wastewater and aquatic environments, the newly isolated yeast *S. halophilus* SSA1575, could potentially be utilized for bioremediation of azo dye wastewaters at a high concentration of azo dyes. Singh^34^ reported that the dye concentrations in textile wastewater generally range between 10 and 200 mg L^−1^.

**Table 3:** Comparison of the promising feasibility of *S. halophilus* SSA1575 on decolorization efficiency with other halotolerant yeast strains reported in literature.

Strain	Dye concentration (mg L^−1^)	NaCl concentration (g L^−1^)	Maximum decolorization (%)	Time (h)	References
*Pichia occidentalis* G1	50	30	98	16	^15^
*Scheffersomyces spartinae* TLHS-SF1	80	⩽ 30	90	16	^16^
*Galactomyces geotrichum* GG	100	⩽ 40	92	10	^17^
*Cyberlindnera samutprakarnensis* S4	50	30	97	18	^18^
*S. halophilus* SSA1575	50	5	100	6	This study
*S. halophilus* SSA1575	50	20	98.71	12	This study
*S. halophilus* SSA1575	50	40	98	24	This study
*S. halophilus* SSA1575	50	50	92	24	This study

### Salt concentration

**Figure 4:**
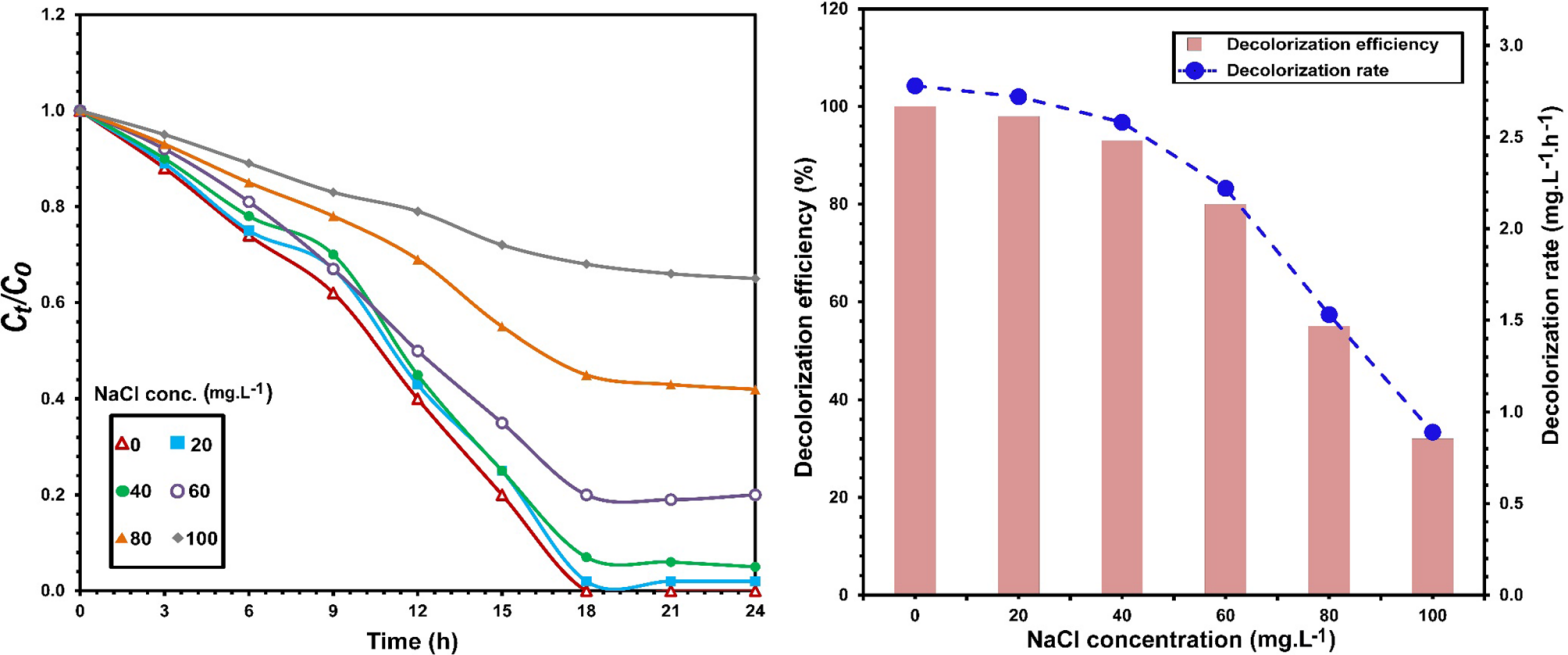
Influence of salt concentration on the decolorization of RB5 by *S. halophilus* SSA-1575, inset with the corresponding decolorization efficiency and decolorization rate.

The efficiency of *S. halophilus* SSA1575 to decolorize 50 mg L^−1^ RB5 in the presence of a high salt concentration was evaluated (Fig. 4). After incubation for 12 h, nearly complete decolorization of RB5 by the SSA1575 strain was observed under the effect of 20 g L^−1^ NaCl. However, the RB5 decolorization efficiency reached above 90% at NaCl concentration of up to 50 g L^−1^ after 18–24 h. A further increase in the salt concentration above 50 g/L showed significant decrease in the decolorization efficiency of RB5 by the yeast strain SSA1575, reaching less than 29% when the concentration NaCl was 100 g L^−1^. On the other hand, the growth rate of strain SSA1575 at salt concentration (0–50 g L^−1^ NaCl) was significantly better than any higher salinity when beyond 50 g L^−1^ (data not shown). As a result, the newly isolated yeast strain *S. halophilus* SSA-1575 is identified as a halotolerant yeast instead of a halophilic one^35^. Our findings are in agreement with halotolerant yeasts that have been reported in references for azo dye decolorization^15–18^. Thus, it could be concluded that *S. halophilus* SSA1575 could be an efficient halotolerant azo-degrading yeast with great potential for disposal of azo dye wastewaters with the salt contaminant at a high concentration.

### pH and temperature

**Figure 5:**
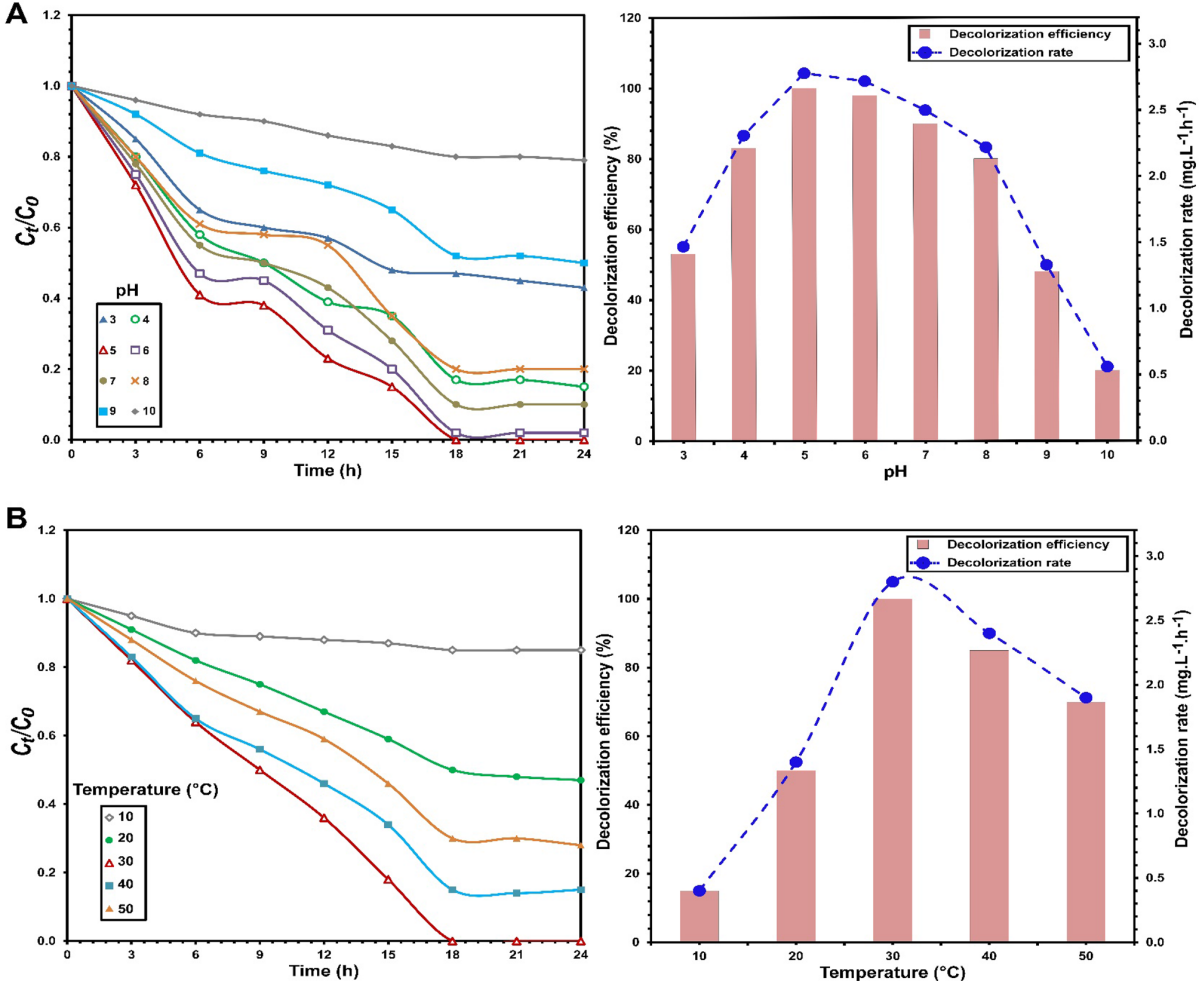
Influence of pH (**A**) and environmental temperature (**B**) on the decolorization of RB5 by *S. halophilus* SSA-1575, inset with the corresponding decolorization efficiency and decolorization rate.

With regard to pH and temperature parameters, their effects on dye decolorization performance driven by *S. halophilus* SSA1575 were evaluated (Fig. 5). As depicted in Fig. 5A, the influence of pH variation differed. The decolorization efficiency of RB5 reached 100% at pH 5 after incubation for 18 h with a maximum dye removal rate of 2.78 mg L^−1^ h^−1^, whereas a significant decrease in decolorization efficiency was observed at strongly acidic or alkaline pH conditions (*p* = 0.003). Hsueh and Chen^36^ reported that the diminishment of dye decolorization at acidic pH, was probably due to the formation of protonated azo dyes that lead to change in the chemical structure of the dye. Hence, microorganism could not recognize the modified dye. However, over 50% RB5 decolorization by *S. halophilus* SSA1575 was still observed at a strongly acidic pH value (pH 3.0) with a maximum dye removal rate of 1.47 mg L^−1^ h^−1^, which might be meaningful in the treatment of azo dyes at acidic conditions. Our findings are in agreement with Du et al.^37^.

To evaluate the influence of environmental temperature, which has an effect on enzymatic activities related to dye decolorization, the effect of temperature on RB5 decolorization by *S. halophilus* SSA1575 was investigated (Fig. 5B). Complete decolorization was observed at 30 °C after incubation for 18 h with a maximum dye decolorization rate of 2.8 mg L^−1^ h^−1^, but nearly 100% and over 85% decolorization efficiency of RB5 were achieved at 40 and 50 °C after 24 h. Significant sharp decline in decolorization efficiency was also observed at 10 °C. Our findings are in agreement with Du et al.^37^ and Tan et al.^38^ who found that low temperatures could significantly inhibit dye decolorization. Comparatively, in this study, *S. halophilus* SSA1575 still showed a potential decolorizing capability at higher temperatures up to 50 °C, suggesting its valuable potential in the bioremediation of azo dyes.

### Carbon and nitrogen sources

**Figure 6:**
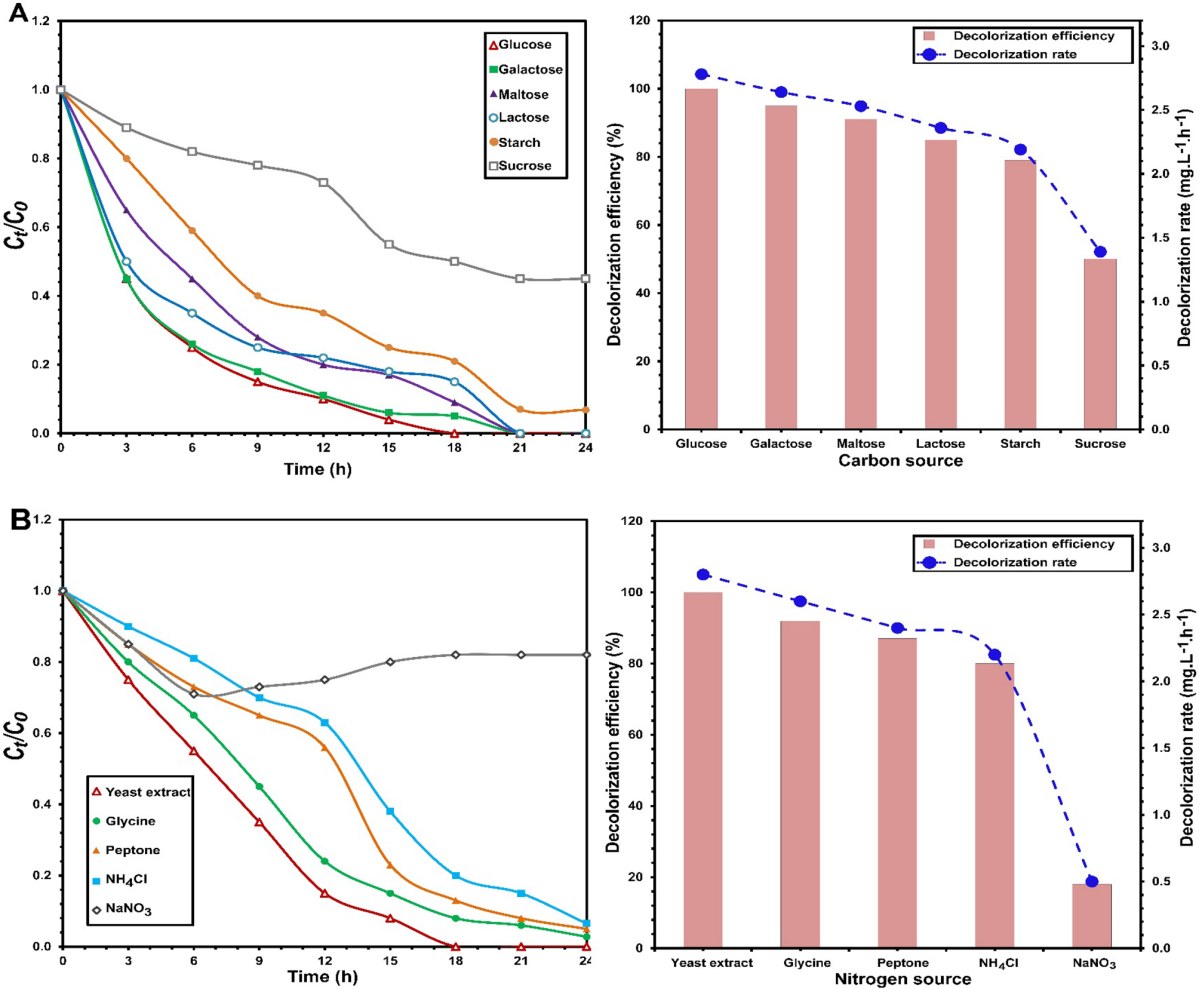
Influence of various carbon sources (**A**) and nitrogen sources (**B**) on the decolorization of RB5 by *S. halophilus* SSA-1575, inset with the corresponding decolorization efficiency and decolorization rate.

The performance of *S. halophilus* SSA1575 on decolorization efficiency of RB5 in the presence of various carbon and nitrogen sources was also studied (Fig. 6). Typically, azo dyes have low carbon content, which makes biodegradation of azo dyes extremely difficult without any supplemental sources of carbon or nitrogen^37,39,40^. Except sucrose, which was slightly inhibited dye decolorization, other added carbon sources (glucose, galactose, maltose, lactose and starch) could significantly (*p* < 0.001) enhance RB5 decolorization by *S. halophilus* SSA1575 (Fig. 6A). Of these, glucose was the optimal carbon source, showing complete decolorization of RB5 after 18 h with a maximum dye decolorization rate of 2.78 mg L^−1^ h^−1^, which was probably due to the easy metabolism of glucose that was taken up inside the yeast cells^41^. Similar to the carbon sources, most tested nitrogen sources could also significantly (*p* < 0.001) enhance RB5 decolorization (Fig. 6B). Yeast extract was the best nitrogen source, showing complete decolorization of RB5 after 18 h with a maximum dye decolorization rate of 2.8 mg L^−1^ h^−1^. However, the addition of sodium nitrate (NaNO_3_) strongly inhibited RB5 decolorization. Similar results were reported previously^37^.

### Repeated additions of dye aliquots

To understand the capability of *S. halophilus* SSA1575 to decolorize repeated additions of dye aliquots well, 50 mg L^−1^ RB5 was tested in decolorization experiments. The decolorization of the first dye aliquot reached 100% within 42 h. The second dye aliquot was also decolorized (100%) by *S. halophilus* SSA1575 within the next 36 h. However, the third and fourth RB5 aliquots, which were subsequently added, showed complete decolorization within 30 and 24 h of addition, respectively. Further added dye aliquots (fifth, sixth and seventh) revealed also complete decolorization of RB5 only within 18 h. Clearly, the reduction in time required for decolorization of RB5 by *S. halophilus* SSA1575 in the next cycle was probably due to acclimatization of this yeast strain to the particular azo dye^11^. Therefore, the ability of *S. halophilus* SSA1575 to decolorize repeated additions of dye aliquots within a short time is significant for its commercial application.

### Toxicity assessment

**Figure 7:**
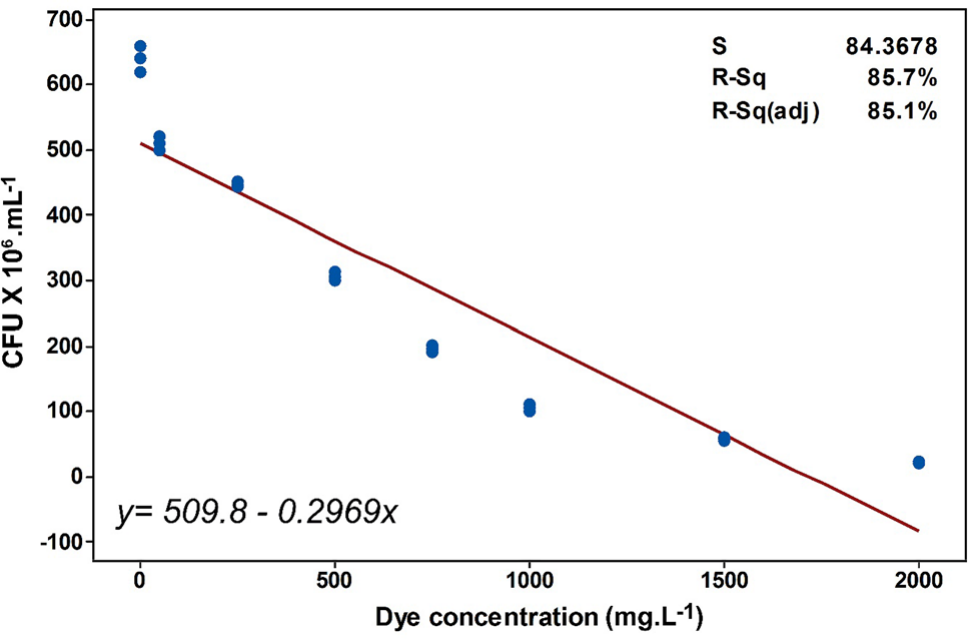
Toxicity assessment. Microbial toxicity of biotransformation metabolites based on the viable colony counting bacterial cells, *Sinorhizobium meliloti*. *p*-value < 0.05 is considered significant.

Some of the intermediate metabolites, produced from dye degradation, could accumulate in the environment and might be more toxic than the original dye^42^. Hence, phytotoxicity and microbial toxicity assays were conducted finally, to evaluate the safety of the metabolic intermediates produced after the decolorization of RB5 by *S. halophilus* SSA1575. Phytotoxicity, which becomes a more prevalent assay due to being less expensive and easier than other methods, was also performed to evaluate the toxicity of the untreated and treated dye. Therefore, phytotoxicity of RB5 and its extracted metabolites formed after degradation by *S. halophilus* SSA1575, was evaluated using *Sorghum vulgare* and *Phaseolus mungo* seeds (Table 4). The RB5 (100 ppm) showed a higher inhibitory effect on the plumule and radicle lengths of both plant seeds when compared to extracted metabolites obtained after dye degradation. The RB5 solution exhibited 50 and 60% germination inhibition in the case of *S. vulgare* and *P. mungo* seeds respectively, when compared with metabolites (Table 4). These results are in agreement with Guo et al.^17^ and Saratale et al.^39^ who reported that the metabolites of azo dyes exhibited lower toxicity when compared with the original dyes. On the other hand, the microbial toxicity based on the number of viable cells of *Sinorhizobium meliloti* was also performed (Fig. 7). The viability of *S. meliloti* cells was significantly decreased along with an increased RB5 concentration, as revealed from the negative linear correlation between the number of colonies and the dye concentration (*p* < 0.001; r =− 0.9). The linear regression model indicated that the RB5 dye concentration has a significant predicted impact on cell viability that can account for 85.7% of explained variability in the living cell count. This finding was also consistent with previous reports^43^. It can be concluded that the results of toxicity evaluations indicate a capability of the newly isolated halotolerant *S. halophilus* SSA1575, in converting the recalcitrant azo dye RB5 into some non-toxic metabolites. Hence, this yeast strain can be safely implemented in a bioremediation process, particularly for industrial wastewater containing high-salt azo dyes.

**Table 4:** Phytotoxicity assessment of RB5 and its metabolites formed after degradation by *S. halophilus* SSA-1575.

Parameters	Distilled water	RB5	Extracted metabolites
***Sorghum vulgare***			
Germination (%)	100	50	100
Plumule (cm)	7.4 ± 0.11	3.1 ± 0.13*	6.2 ± 0.09**
Radicle (cm)	3.5 ± 0.09	1.2 ± 0.07*	2.4 ± 0.07**
***Phaseolus mungo***			
Germination (%)	100	40	100
Plumule (cm)	10.7 ± 0.40	2.3 ± 0.33*	7.5 ± 0.35**
Radicle (cm)	5.3 ± 0.11	0.7 ± 0.15*	3.1 ± 0.15**

Values are mean of triplicate ± SD.

*Significantly different from the seeds germinated in distilled water at *p* < 0.05 by one-way ANOVA with Tukey–Kramer multiple comparisons test.

**Significantly different from the seeds germinated in RB5 dye at *p* < 0.05 by one-way ANOVA with Tukey–Kramer multiple comparisons test.

### Decolorization mechanism in terms of enzymatic analysis and byproducts detection

As depicted in Table 5, the activities of three oxidases (Lac, LiP and MnP) and two reductases (NADH-DCIP reductase and azoreductase) were analyzed over a period of RB5 decolorization by *S. halophilus* SSA1575. Song et al.^18^ reported that NADH-DCIP reductase and azoreductase enzymes were primarily responsible for the cleavage of –N=N– of azo dyes, while Lac, MnP and LiP were involved in further steps of the biodegradation intermediates, such as amines. In this study, the oxidases and reductases were measured under both low (0 g L^−1^ NaCl) and high (40 g L^−1^ NaCl) salt concentrations. The activity of NADH-DCIP reductase was significantly induced (*p* < 0.005) intracellularly and extracellularly under high-salt concentration by 102% and 94%, respectively over the control (Table 5). On the other hand, the activity azoreductase was not detected under high salinity, which suggested that the NADH-DCIP reductase was probably involved in the first step of RB5 degradation. The activities of LiP, MnP and Lac, which were probably involved in further biodegradation steps of RB5, were detected only intracellularly under both low- and high-salt concentrations. However, these activities were significantly decreased under high salinity during the degradation processing when compared with their controls (*p* < 0.001). As shown in Table 5, the activities of LiP and Lac were detected as crucial intracellular oxidases of the yeast *S. halophilus* SSA1575, with relatively higher activities when compared with the activity of MnP. Song et al.^15^ reported the possible inhibitory effect at a high salt concentration on ligninases (oxidases). Clearly, *S. halophilus* SSA1575 could efficiently decolorize RB5 due to the unique enzymatic system involved.

**Table 5:** Extracellular and intracellular enzymes’ activities produced after decolorizing 50 mg L^−1^ RB5 by the halotolerant yeast strain SSA-1575.

Enzyme	Control		High salinity	
	Intracellular	Extracellular	Intracellular	Extracellular
Lac^a^	0.367 ± 0.002	ND	0.083 ± 0.001*	ND
LiP^a^	0.452 ± 0.008	ND	0.283 ± 0.001*	ND
MnP^a^	0.171 ± 0.006	ND	0.023 ± 0.0002*	ND
NADH-DCIP reductase^b^	20.7 ± 0.04	12.93 ± 0.05*	41.74 ± 0.67*	25.03 ± 0.11*
Azoreductase^c^	2.83 ± 0.63	0.11 ± 0.015	ND	ND

Values are mean of triplicates ± SD. Significantly of enzyme activities after dye decoloration differ from target dye (control) at * p < 0.05 using one-way (ANOVA) with Tukey–Kramer comparison test. *ND* not detected.

^a^Activity measured by U/min/mg protein.

^b^Activity measured by µg of DCIP reduced/min/mg protein.

^c^Activity measured by µg of methyl red reduced/min/mg protein.

**Figure 8:**
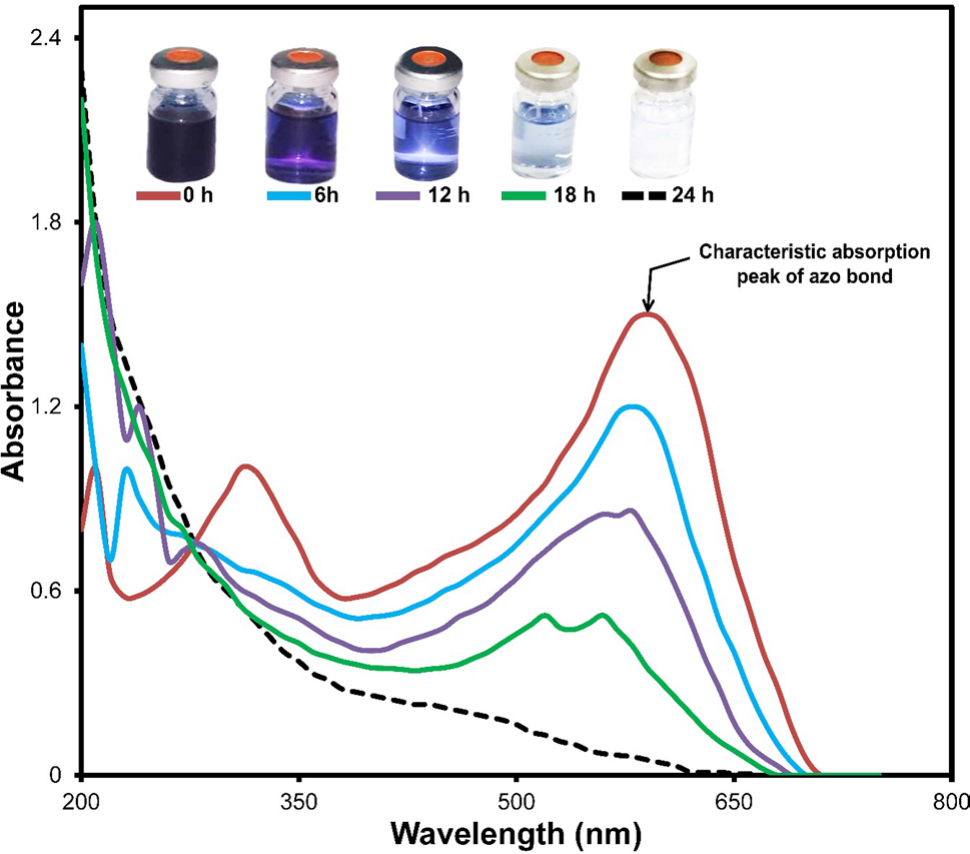
UV–vis spectra of 50 mg L^−1^ RB5 before and after decolorization by *S. halophilus* SSA1575.

To understand well the possible mechanism of dye decolorization, the byproducts made by the halotolerant yeast strain *S. halophilus* SSA1575 during RB5 degradation were analyzed via UV–Vis spectroscopy, Fourier transformed infrared spectroscopy (FTIR) and Mass Spectrometry techniques. The changes in the UV–vis absorption spectra of RB5 solution (with an initial dye concentration of 50 mg L^−1^ and salt concentration of 40 g L^−1^ NaCl) treated with *S. halophilus* SSA1575 within 24 h were presented (Fig. 8). It has been reported that the intensity of –N=N– bond is proportional to the concentration of azo dye in solution^1^. UV–vis absorption spectra of RB5 exhibited a strong absorption at λ_max_ = 595 nm, which originated from the conjugated aromatic rings connected by the –N=N– bond. The other observed two peaks at 203 and 310 nm were attributed to the benzene and naphthalene rings of the dye^44^. Azo dye could be decolorized by microbial cells through biodegradation or adsorption. In the biodegradation process, either the major absorption peaks in the visible light region of the spectrum disappears completely or a new peak appears. However, in the case of the adsorption process, the UV–vis absorption peaks decrease approximately in proportion to each other^45,46^. As depicted in Fig. 8, the absorbance observed at 595 nm (at the beginning of the dye decolorization process) was decreased with a small shift towards a shorter wavelength, where two peaks at 562 and 530 nm were observed and probably attributed to the formation of other metabolites, such as quinone and benzene molecules^11,20,43^. On the other hand, the peaks at 211, 230 and 278 nm disappeared, while a new peak (206 nm) was observed. As the reaction progressed, the intensity of the absorption peak (595 nm) decreased to almost zero after 24 h of RB5 decolorization by *S. halophilus* SSA1575, which was verified from the fully transparent solution (Fig. 8). Clearly, such changes in the absorbance were probably attributed to the changes in the molecular structure of RB5 (deconstruction of its primary chromatophores), indicating degradation of the –N=N– bond, formation of amino groups and other intermediate metabolites, and deconstruction of RB5 during the dye decolorization process^7,47^.

**Figure 9:**
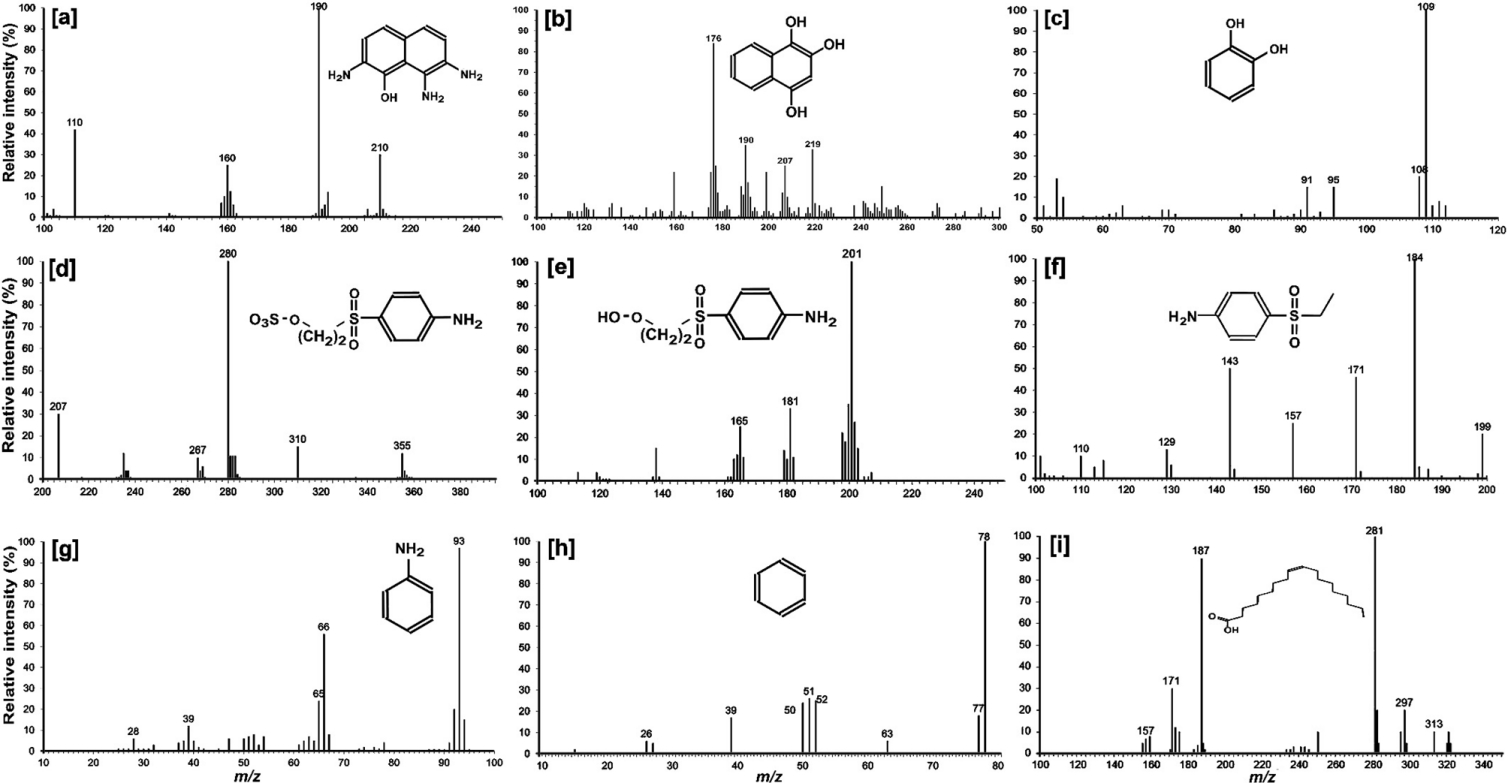
The intermediate metabolites of RB5 degradation identified by GC–MS analysis.

The nature of the degradation product of RB5 by *S. halophilus* SSA1575 was verified from FT-IR analysis. Comparison of the FT-IR spectrum of the original dye molecule (RB5) with metabolic products extracted after the complete decolorization process clearly indicated significant changes and biodegradation of the RB5 molecule, which were evidenced from the formation of some new peaks as well as the disappearance of some initial peaks (Table 6). In fact, –N=N–, NH_2_, aromatic amines, C–C linkages showed a cleavage with a prolonged decolorization reaction time and cleavage of azo bonds in RB5^48^. The decrease in the intensities of the peaks at the low-frequency region of spectra (620–850 cm^−1^) suggested the fission of aromatic rings^49^. As a result, it is very clear that the molecular structure of RB5 was significantly deconstructed during the decolorization process, by the newly isolated yeast strain *S. halophilus* SSA1575. To further propose the possible degradation pathway of RB5 by SSA1575, possible metabolites were detected using Mass Spectrometry technique. The structures of nine possible decolorization byproducts could be drawn from the mass spectra and *m*/*z* values as depicted in Fig. 9.

**Table 6:** Absorption spectra of RB5 before and after its decolorization.

Wave number (cm^−1^)	Assignment	Before decolorization	After decolorization
3,497	N–H stretching vibration in amides	+	−
1,730	COOH stretching	−	+
1,670	C = C aromatic stretching vibration	+	−
1,609, 1,551	N = N stretching	+	−
1,487	Ring vibrations	+	−
1,422	Benzene ring	+	+
1,335	NO_2_ stretching	+	−
1,170	Sulfone SO_2_ stretching	+	+
1,045, 1,086, 1,202	C–N stretching confirm the azo nature of dye	+	−
845	C–H deformation	+	−
783	N–O stretching	+	−
699, 623	–C–S-stretching vibration	+	−

** + **: present, −: absent.

**Figure 10:**
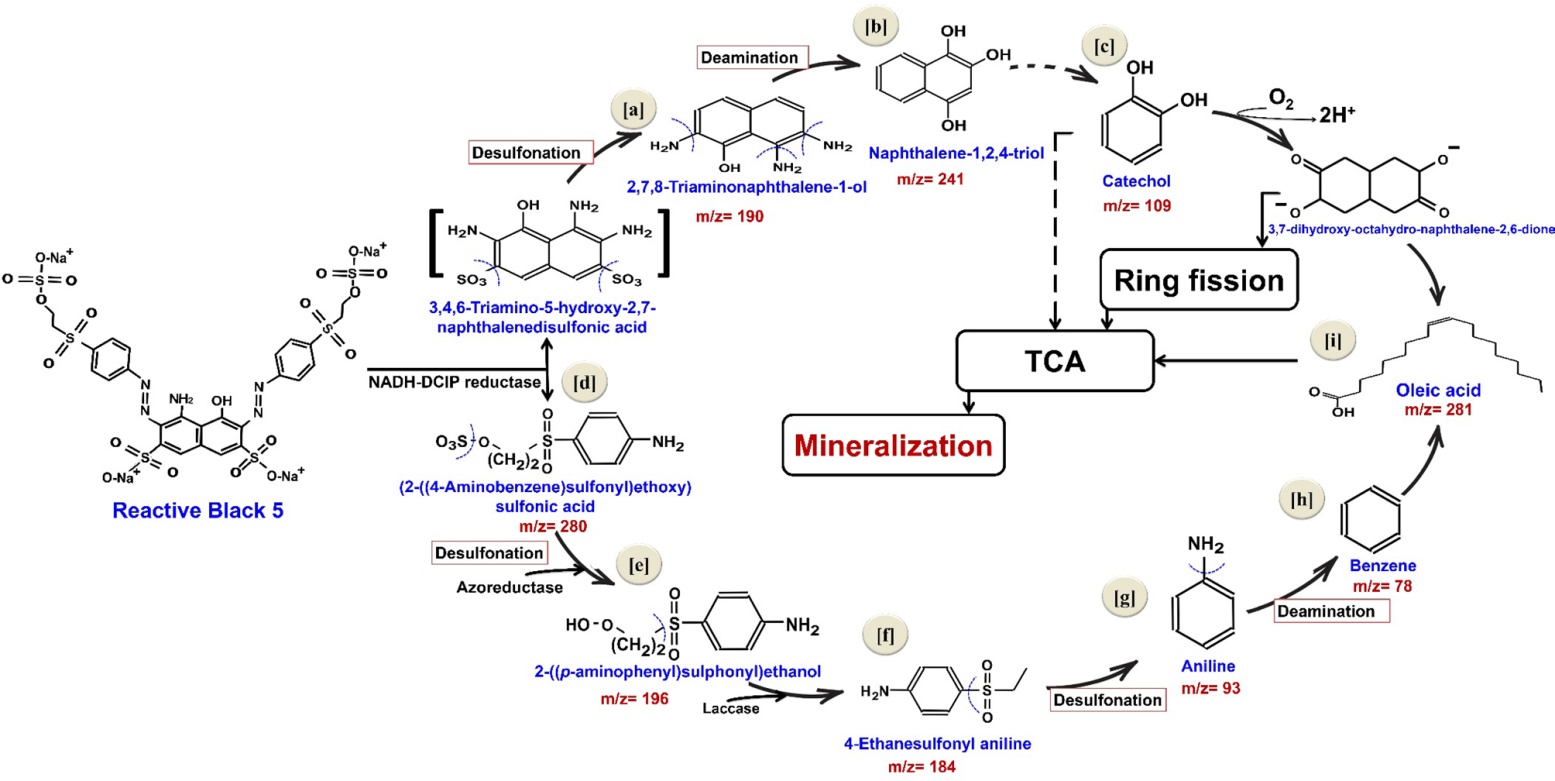
Proposed pathway for the degradation of RB5 by *S. halophilus* SSA1575.

Figure 10 depicts the possible pathway of RB5 biodegradation by *S. halophilus* SSA1575. The asymmetrical reduction of azo bonds of RB5 by the action of NADH-DCIP reductase was considered as the first step towards bye degradation, resulting in the formation of amines (2-((4-aminobenezene)sulfonyl)ethoxy)sulfonic acid [d] and 1,2,7-triamino-8-hydroxy-3,6-naphthalinedisulfonate (TAHNDS). However, TAHNDS is an unstable compound and it might be rapidly transformed to other smaller intermediate metabolites^16,18,50,51^, such as 2,7,8-Triaminonaphthalen-1-ol [a]. Subsequently, both of the compounds [a] and [d] were probably further oxidatively transformed into smaller compounds and may eventually be mineralized as depicted in Fig. 10. The intermediate metabolite 2,7,8-Triaminonaphthalen-1-ol [a] was probably deaminated into naphthalene-1,2,4-triol [b], then transformed to catechol [c], which might be cleaved oxidatively into aliphatic metabolites via the cis-muconic acid pathway^52^ followed by the TCA cycle and finally leading to complete mineralization of RB5 by *S. halophilus* SSA1575. On the other hand, (2-((4-aminobenezene)sulfonyl)ethoxy)sulfonic acid [d] was probably further oxidatively transformed into 2-((4-aminophenyl)sulfonyl)ethanol [e], 4-ethanesulfonyl aniline [f] and aniline [g] through desulfonation. The later metabolite was probably then deaminated into benzene [h]. According to Gutiérrez et al.^53^, benzene was successfully used as a precursor for synthesizing a saturated fatty acid by *Rhodococcus* sp. strain 33. Finally, *cis*-9-octadecenoic acid [i] was probably mineralized. Overall findings suggested the efficiency of *S. halophilus* SSA1575 to decolorize and degrade RB5 under high salinity, suggesting that this newly isolated halotolerant yeast strain would have a high potential in the bioremediation of azo dye wastewater with a high load of salts.

## Methods

### Reagents and culture media

Five Azo dyes (Sigma-Aldrich, St. Louis, USA) were used in this study. The molecular structures and absorption wavelength of these dyes were shown in Fig. S1. Other chemical reagents were purchased from Sinopharm Chemical Reagent Co., Ltd. (China). Biochemical reagents and Dr. GenTLER (from Yeast) High Recovery were purchased from TaKaRa Biotechnology Co., Ltd. (Japan). Yeast Malt Extract (YME) consisted of (g/L): 3.0 yeast extract; 5.0 peptone 3.0 malt extract and 10 glucose. Minimal Saline (MS) medium consisted of (g/L): 0.05 CaCl_2_, 0.05 MgSO_4_, 1.0 KH_2_PO_4_, 0.2 (NH_4_)_2_SO_4_ and 20 NaCl was used, containing 10 glucose as carbon source.

### Isolation and identification of azo-decolorizing yeasts

A total of seven novel yeast species or strains which could efficiently decolorize azo dyes under high salt conditions were successfully isolated from the gut of *R. chinenesis* as per our previously described method^13^. Phenotypic characterization of the highest decolorization efficiency performed by a yeast strain in a relatively short period of time was first examined following standard methods^54,55^. To identify the yeast genetically, genomic DNA was extracted using Dr. GenTLER (from Yeast) High Recovery according to the manufacturer's instructions. The 26S rDNA gene was amplified by PCR with the primers NL1/NL4^3,13^. Then the purified PCR products were sequenced by Sangon Biotech (Shanghai, China). All sequences were submitted to GenBank (accession numbers in Table 2) and aligned with sequences available at BLAST-n (https://www.ncbi.nlm.nih.gov/BLAST/). The evolutionary history was inferred using the UPGMA method^56^. The evolutionary distances were computed using the Kimura 2-parameter method^57^ and are in the units of the number of base substitutions per site. The rate variation among sites was modeled with a gamma distribution (shape parameter = 2). All positions containing gaps and missing data were eliminated. Evolutionary analyses were conducted in Evolutionary Genetics Analysis (MEGA) version 7^58^.

### Decolorization experiments

The Aqueous solutions (0.1 g) of RB5 as well as other azo dyes were prepared by dissolving the dye tested at the desired concentration in distilled water to prepare 500 mL of solution. Decolorization experiments were conducted in an Erlenmeyer flask (100 mL) containing a volume (5%) of yeast suspension (OD_600_ of 0.2). Before incubation at 30 °C for 24 h under static condition, the dye was added to flasks at a concentration of 50 mg L^−1^. Periodically, a 2 mL sample was withdrawn and centrifuged at 12,000 rpm for 3 min. The clear supernatant was used to measure the concentration of the dye by UV–vis spectrometer (Model Shimadzu-UV2600, Japan) at the absorbance maximum of 650 nm (AzB), 595 nm (RB5), 537 nm (RR120), 592 nm (RB19) and 511 nm (AS-GR). Un-inoculated control was included to compare color loss during the decolorization experiments.

The fastest decolorized dye in a relatively short period of time was selected as the target dye for further investigation of the optimization of dye decolorization. The decolorization performance of RB5 (50 mg L^−1^) by *S. halophilus* SSA1575, was evaluated at varying salt concentrations (10–100 g L^−1^ NaCl with an interval of 10 g L^−1^), dye concentrations (50, 100, 250, 500, 750, 1,000, 1,500 and 2000 mg L^−1^), temperatures (5–50 °C with an interval of 5 °C) and initial pH (3.0–10.0 with an interval of 1.0). The effect of different carbon and nitrogen sources (2% each) on decolorization efficiency of RB5 by *S. halophilus* SSA1575 was also studied using glucose, galactose, sucrose, maltose, lactose and starch as carbon sources, while yeast extract, peptone, NaNO_3_, NH_4_Cl and glycine were used as nitrogen sources. Further experiments were also performed to evaluate the performance of *S. halophilus* SSA1575 to decolorize repeated additions of 50 mg L^−1^ RB5 aliquots.

Decolorization of mixture of azo dyes were calculated following the ADMI protocol^59^. ADMI removal percentage is defined as the ratio between the ADMI removal value after a particular contacting time (*t*) and the ADMI value at initial concentration. Four combination mixtures containing two dyes (RB5 and RB19), three dyes (RB5, RB19 and RR120), four dyes (RB5, RB19, RR120 and AzB) and five dyes (RB5, RB19, RR120, AzB and AS-GR) were used at a concentration of 0.2 g L^−1^. All decolorization experiments were performed in triplicates.

### Analytical methods

The percentage of decolorization of dye mixtures was calculated following the Standard Method^60^. To identify possible metabolites as well as to predict possible mechanisms during decolorization process of the target dye by *S. halophilus* SSA1575, UV–vis spectroscopy in the region spectrum of 200–700 nm, FTIR in the IR region of 400–4,000 cm^−1^, and Mass Spectrometry were used under the operation conditions reported earlier^61,62^.

### Toxicity assessment

#### Phytotoxicity assay

Phytotoxicity was determined in this study based on the inhibition of seed germination using the most-used species *S. vulgare* (monocot seeds) and *P. mungo* (dicot seeds)^63^. The ethyl acetate products of RB5 degradation were dissolved in sterile distilled water to make a final concentration of 100 mg/L. Twenty seeds for each plant set were separately watered with a 5 mL solution of RB5 (100 ppm) or the decolorization metabolites. The seeds watered by distilled water were used as a control set. The changes in radical and plumule lengths were measured after 7 days of germination.

#### Microbial toxicity assay

This assay was performed using the model soil bacterium *S. meliloti* that establishes nitrogen-fixing symbiosis with alfalfa^64^. In this test, the products of dye biodegradation were assessed based on the viable colony counting of *S. meliloti* cells. A volume of 5% freshly cell suspension (OD_600_ of 0.2) was inoculated into 5 mL of sterilized yeast mannitol broth at different concentrations of the dye decolorized medium which was previously filtered (0.2 µm). The viable bacterial colonies were counted using the pour plate technique.

### Enzyme assay

Extracellular and intracellular enzyme solutions of *S. halophilus* SSA1575 cells before and after the target dye decolorization for 24 h in a liquid medium containing 50 mg L^−1^ RB5 and 40 g L^−1^ NaCl were prepared following the method described previously^65^. Activities of reductase enzymes including azoreductase and nicotinamide adenine dinucleotide-dichlorophenol indophenol (NADH-DCIP) reductase were monitored as described by Song et al.^15^. Activities of oxidative enzymes such as LiP, MnP and Lac were also determined as reported earlier by Ali et al.^65^ . All enzyme assays were carried out spectrophotometrically at room temperature. A blank contained all components except the enzyme. One unit of enzyme activity was defined as the amount of substrate consumed or product generated per milligram protein per minute. Protein concentration of the enzyme solution was monitored using the Bradford method^66^.

### Statistical analysis

Results were statistically analyzed using Minitab 17.1.0.0 and SigmaPlot Software 12.5.0.38. The normality of data was estimated by the Shapiro Wilk test. One-, two- or three-way ANOVA test used with multiple comparisons by Tukey methods. Simple linear regression analysis was performed to estimate the effect of the dye and its biotransformed metabolites on the viability of cells using a regression equation for prediction. The *p*-value < 0.05 is considered significant.

## Supplementary information

Supplementary information.

## Data Availability

All data are available upon request.
