# Supplementary material for: Ecofriendly biodegradation of Reactive Black 5 by newly isolated Sterigmatomyces halophilus SSA1575, valued for textile azo dye wastewater processing and detoxification
Source: Sci Rep. 2020 Jul 23;10:12370. doi: 10.1038/s41598-020-69304-4 (PMC7378048; doi:10.1038/s41598-020-69304-4)
Supplement: Supplementary file 1 — Supplementary information. [file 41598_2020_69304_MOESM1_ESM.docx]

**Ecofriendly biodegradation of Reactive Black 5 by newly isolated *Sterigmatomyces halophilus*** **SSA1575, valued for textile azo dye wastewater processing and detoxification**

Rania Al-Tohamy^1^, Jianzhong Sun^1*^, Mervat Fareed^2^, El-Refaie Kenawy^3^, Sameh Samir Ali^1,4*^

^1^Biofuels Institute, School of the Environment and Safety Engineering, Jiangsu University, Zhenjiang, 212013, China

^2^Department of Home Economic, Faculty of Specific Education, Tanta University, Tanta, Egypt

^3^Polymer Research Group, Department of Chemistry, Faculty of Science, Tanta University, Tanta, 31527, Egypt

^4^Botany Department, Faculty of Science, Tanta University, Tanta, 31527, Egypt

*Corresponding authors at: Biofuels Institute, School of the Environment and Safety Engineering, Jiangsu University, Xuefu Rd. 301, 212013, Zhenjiang, China.

**E-mail**: [jzsun1002@ujs.edu.cn](mailto:jzsun1002@ujs.edu.cn) (J. Sun); [samh@ujs.edu.cn](mailto:samh@ujs.edu.cn), [samh_samir@science.tanta.edu.eg](mailto:samh_samir@science.tanta.edu.eg) (S.S. Ali).


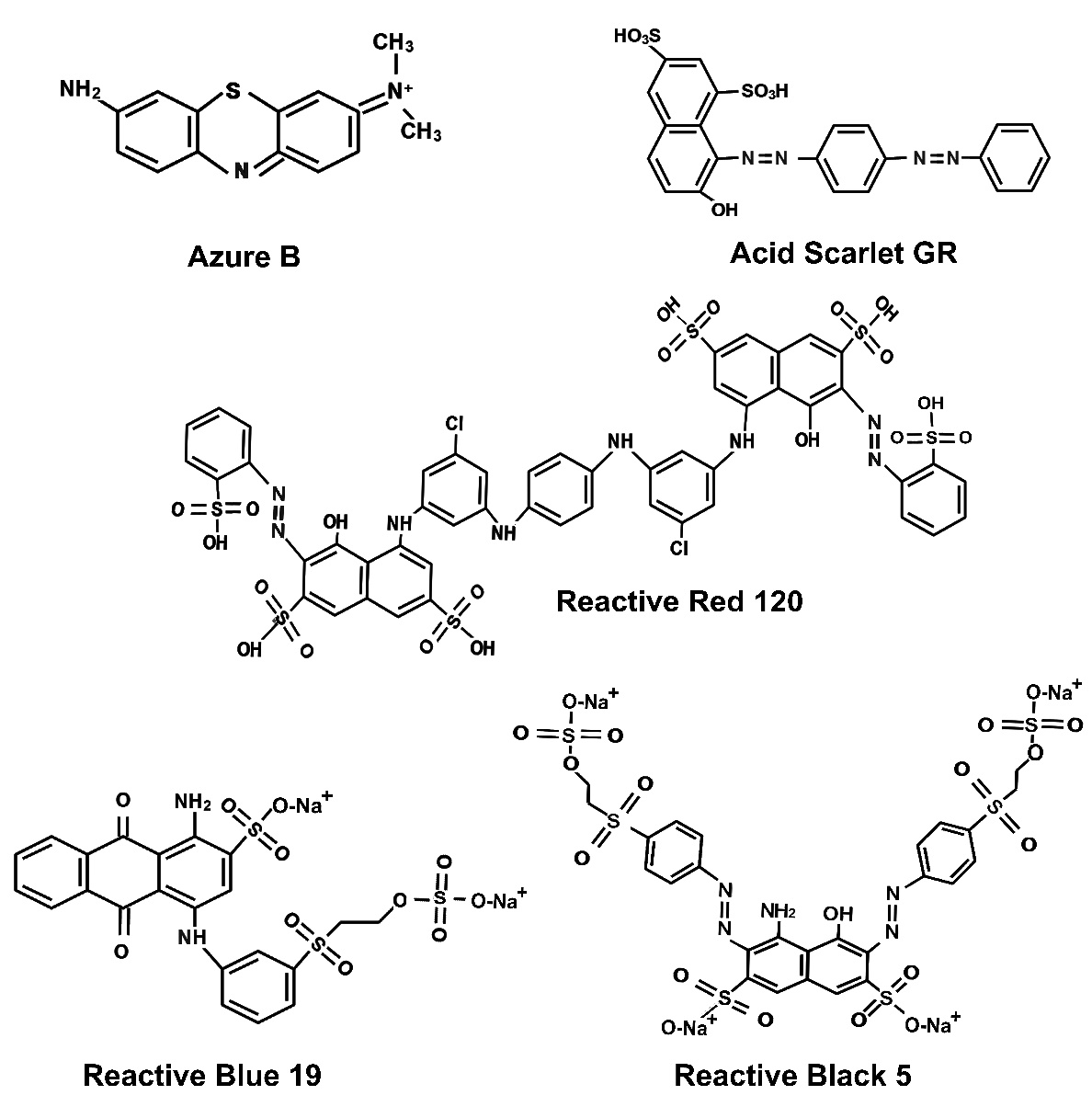


**Fig. S1.** Molecular structures of azo dyes used in this study.
